# Approved and Emerging Disease Modifying Therapies on Neurodegeneration in Multiple Sclerosis

**DOI:** 10.3390/ijms21124312

**Published:** 2020-06-17

**Authors:** Madeline Bross, Melody Hackett, Evanthia Bernitsas

**Affiliations:** Department of Neurology, Wayne State University School of Medicine, Detroit, MI 48201, USA; mbross@med.wayne.edu (M.B.); melodyhackett@wayne.edu (M.H.)

**Keywords:** neurodegeneration, multiple sclerosis, disease modifying therapies, brain atrophy, demyelination

## Abstract

Multiple sclerosis (MS) is an autoimmune, chronic, progressive disease leading to a combination of inflammation, demyelination, and neurodegeneration throughout the central nervous system (CNS). The outcome of these processes can be visualized in magnetic resonance imaging (MRI) scans as brain atrophy, or brain volume loss (BVL), as well as lesions, “black holes” and spinal cord atrophy. MRI outcomes such as BVL have been used as biomarkers of neurodegeneration and other measures of MS disease progression in clinical research settings. Several FDA-approved medications seek to alleviate disease progression by reducing the impact of such factors as demyelination and neurodegeneration, but there are still many shortcomings that current clinical research aims to mitigate. This review attempts to provide an overview of the FDA-approved medications available for treating multiple sclerosis and their effect on neurodegeneration, measured by BVL.

## 1. Introduction

Multiple sclerosis (MS) is a chronic demyelinating and degenerative immune-mediated condition of the central nervous system (CNS) characterized by a wide spectrum of disease evolution, from focal inflammation to neuronal death, axonal and myelin loss, and failure of CNS repair mechanisms to restore the damage [1]. These underlying mechanisms result in various MS symptoms, consisting of muscle weakness, fatigue, imbalance, and increased ambulatory difficulty [2].

Brain atrophy, or brain volume loss (BVL), may be present in early stages of MS. BVL becomes more prominent in progressive stages and has been used as a biomarker for neurodegeneration [3]. Brain atrophy can be visualized in magnetic resonance imaging (MRI) scans and is being used as a measure of disease progression in MS, mainly in clinical research and not as a part of routine clinical practice [4,5]. It is well established that the rate of BVL occurs faster in MS patients than in healthy individuals. In MS, BVL is estimated to be between 0.5% and 1.35% per year, with an average of 0.7% per year, compared to 0.2% to 0.4% in age-matched healthy controls [6,7].

Brain atrophy presents in early stages of MS and is associated with physical and cognitive disability [8]. While in most cases increased BVL or a high rate of BVL can indicate a poor prognosis, it is difficult to generalize all such instances under the same trend [9]. Several studies have used other imaging biomarkers for axonal loss, such as thalamic volume, gray matter fraction, corpus callosum area, “black holes,” and spinal cord atrophy to predict the course of the disease as is seen using clinical measures [3,10,11]. However, BVL is the preferred biomarker to assess neurodegeneration and even though its use has not been implemented in common clinical practice, it is used mainly in the research setting. Some of the currently available treatment options aim to manipulate this pro-inflammatory response by targeting the cells responsible for mediating these mechanisms [12]. It is hypothesized that prior inflammation makes it more likely for demyelination and neurodegeneration to then occur [13].

## 2. Inflammation

Inflammation is more prominent in the early stages of MS and occurs when activated microglia and macrophages target specific cell types in the CNS, destroying oligodendrocytes and leading to demyelination of axons [14]. The role of T- and B-lymphocytes is critical in the inflammatory process. MS was thought to be mainly a T-cell disease, as the pro-inflammatory Th1 and Th17 lymphocytes were found within the brain and active plaques in people with MS. These types of cells are sequestered within lymph nodes under fingolimod treatment and in the peripheral blood after treatment with natalizumab. More recently the role of B-lymphocytes was investigated and found to be prominent. B-lymphocytes produce autoantibodies, increase secretion of pro-inflammatory cytokines, and decrease production of regulatory cytokines; all these factors promote and perpetuate inflammation. Inflammatory lesions in the brain and spinal cord can be visualized by imaging modalities such as MRI scans with contrast during the relapses seen in patients with relapsing-remitting MS (RRMS) [15]. Consequently, because of experiencing relapses, patients with RRMS and active secondary progressive MS (SPMS) phenotypes tend to have more enhancing lesions indicating active disease, and higher degrees of inflammation than primary progressive (PPMS) patients [16]. Inflammation may cause axonal injury and loss and contribute to BVL early in the disease process [17]. Relapses are often associated with the development of new lesions and/or growth of currently existing white and gray matter lesions [18]. Inactive lesions in the CNS are also visualized utilizing MRI in MS patients. The number of inactive lesions tends to increase over the course of the disease when remyelination of the affected area does not occur [15].

## 3. Demyelination

Neuroinflammation is the underlying mechanism of demyelinating lesions [19]. Demyelinating lesions with partial preservation of axons is a typical injury seen in MS [10]. The formation of these lesions often activates demyelinating mechanisms where antibodies target and attack a specific antigen or cell type [20]. One of the cell types destroyed are oligodendrocytes, which are responsible for maintaining the myelin sheath surrounding axons and providing nutrients to the neurons [21]. The loss of these cells makes the neurons more susceptible to inflammatory processes and contributes to more myelin damage and loss [22]. The damage to the myelin sheath interferes with normal message transmission. This diminished transmission ability may account for the clinical presentation of MS as well as the severity of the symptoms [23].

## 4. Neurodegeneration: Axonal Injury and Loss

Inflammation, demyelination, and neurodegeneration are certainly linked; however, a causal relation between them is still unclear [24]. It has been found that in active lesions, the level of reactive oxygen species tends to be higher, often leading to oxidative damage and subsequent cell injury [20]. After the loss of the protective myelin sheath, demyelinated axons are thought to become more vulnerable and therefore more likely to sustain cellular damage by pro-inflammatory processes [22].

Neurodegeneration is a complex process where different molecular mechanisms can occur in combination. After the death of oligodendrocytes during active inflammation and the release of iron from them, other mechanisms, such as microglia activation resulting in increased production of free radicals (nitric oxide and glutamate excitotoxicity), and mitochondrial dysfunction may amplify the oxidative injury and lead to worsening neurodegeneration [25,26]. Although primary injury to the axons is the main reason for axonal loss, axons may die in areas far from the lesion due to lack of pre and post-synaptic signals (dying back and Wallerian axonal degeneration). In neurodegeneration and progressive MS, the role of B-lymphocytes appears to be more important than previously accepted, as ocrelizumab, a B-cell depleting disease modifying treatment might affect this process [26,27,28].

Diffuse axonal injury of the normally appearing white and gray matter is a hallmark of progressive forms. Similarly, cortical demyelination, sometimes associated with the presence of B-cell rich ectopic meningeal lymphoid aggregates, may be present early in the disease, but becomes more pronounced in the progressive stages, resulting in irreversible damage [29,30].

## 5. MS Therapies

Regular clinical assessments and MRI scans are the most common way to track MS disease progression. Maintaining records of these measures is also important to determine if and to what extent a disease modifying therapy (DMT) is effectively treating a patient’s MS. A variety of DMTs are currently available via a few different routes of administration (oral, injectable, infusion) for patients with MS. Although it is difficult to generalize a treatment’s true benefits or drawbacks across all patients. A summary table of the available treatment options can be seen in Table 1 below.

## 6. Oral Disease Modifying Therapies

### 6.1. Dimethyl Fumarate

Dimethyl fumarate (DMF), available commercially as Tecfidera, is FDA-approved to treat relapsing forms of MS (including clinically isolated syndrome (CIS), RRMS, and relapsing forms of secondary progressive MS (SPMS)). Dimethyl fumarate is taken orally and requires gradual dose elevation to reach the appropriate treatment dosage. In clinical studies, it has been observed that it activates a pathway which produces cytoprotective enzymes and thereby blocking oxidative processes against the cells [31]. In clinical studies (DEFINE [32] and CONFIRM [33]), it was observed that there was a statistically significant reduction in brain lesion development from the six- month time point until the end of the two-year study. Similarly, fewer gadolinium-enhancing lesions and T1-hypointense lesions formed in that time [32,33]. While in the DEFINE phase 3 there was a significant decrease of brain atrophy from the baseline to 2 years and from month 6 to 2 years [32], reduction in brain atrophy in the CONFIRM did not reach significance [33]. The currently known side effects of dimethyl fumarate include flushing, abdominal pain, diarrhea, and nausea [34].

### 6.2. Fingolimod

Fingolimod (FTY), known commercially as Gilenya, is an FDA-approved DMT for CIS and relapsing MS. It is an oral sphingosine-1-phosphate receptor modulator which subsequently allows it to alter circulating lymphocyte cell groups [35]. In three multicenter, randomized, double-blind, and placebo-controlled phase III clinical trials (FREEDOMS [36], FREEDOMS II [37], and TRANSFORMS [38]), fingolimod reduced the rate of BVL significantly and consistently compared to either placebo or IM interferon beta-1a [39]. Focusing on gray matter Bajrami, et al., showed a favorable effect of fingolimod on the diffuse and local gray matter (thalamus, basal ganglia) over a 2-year period [40]. Interestingly, these results were not reproduced in the INFORMS trial, suggesting that fingolimod has little effect on the pathogenetic mechanisms that lead to BVL and neurodegeneration in PPMS patients, and confirming the different causative and triggering factors behind pathogenesis in RRMS and PPMS [41]. Further observations showed higher BVL in PPMS patients and was associated with a higher EDSS and worsening disability [41,42]. Since there is an increased risk of macular edema and skin cancer with fingolimod treatments, regular check-ins with ophthalmologists and dermatologists are highly suggested [35].

### 6.3. Siponimod

Siponimod, commercially known as Mayzent, is FDA-approved for CIS, RRMS, and active SPMS patients. It is an oral, selective sphingosine-1-phosphate (S1P) 1 and 5 modulator and its structure allows it to easily cross the blood-brain barrier. In the EXPAND clinical trial, there was a significant reduction in brain volume loss in patients on siponimod compared to placebo, consistent during the 24-month duration of the study. As brain atrophy indicates permanent tissue damage, slowing the rate of brain volume loss can be a strong indicator of decreasing the rate of disease progression in SPMS [43]. Some of the currently known risks of taking siponimod are elevated liver transaminase concentrations, bradycardia, macular edema, hypertension, varicella-zoster virus reactivation, and convulsions. Many of these observed adverse effects are consistent with the side effects listed in safety reports of other approved S1P medications [44].

### 6.4. Teriflunomide

Teriflunomide, commercially known as Aubagio, is FDA-approved for CIS and RRMS patients. Its mechanism of action involves inhibition of mitochondrial dihydro-orotate dehydrogenase (DHODH), leading to reduced synthesis of pyrimidine, a necessary component for the proliferation of growing lymphocytes. The lack of DHODH due to teriflunomide, therefore, has a limiting effect on the action of lymphocytes against the nervous system [45]. This mechanism indicates that teriflunomide has a greater direct effect on the immune system rather than the central nervous system [46]. In mouse models, the administration of teriflunomide appeared to be linked with less microstructural damage and lower glutamate levels, which suggests decreased inflammation. The investigators point out that while these outcomes were observed in laboratory research, it may be more difficult to reproduce the same observations in a clinical setting [47]. In the initial analysis, in CIS (TOPIC [46] trial) and RRMS (TEMSO [48] trial), teriflunomide failed to show a significant effect on BVL compared to placebo. However, a posthoc analysis of the phase III TEMSO showed a beneficial effect of teriflunomide 14 mg on white matter tissue loss [49]. A re-analysis of the phase III TEMSO MRI data showed a significant reduction of BVL versus placebo over a 2-year treatment period [50]. Teriflunomide has been found to be safe and tolerable, but side effects may include hair loss or thinning, an increase in liver enzymes, and increased blood pressure, which should be monitored closely [51].

### 6.5. Cladribine

Cladribine (Mavenclad) is FDA-approved to treat RRMS and active SPMS patients. Cladribine is a synthetic deoxyadenosine analog, which interferes with lymphocyte production by inhibiting both B- and T-cell proliferation. Some studies have emphasized its action on T cells more, but its currently accepted mechanism of action involves its depletion of lymphocytes in the body [52]. The data about the effect of cladribine on brain atrophy are controversial [53]. After trying different doses in a large cohort of patients, Filippi, et al., found no significant treatment effect of either dose of cladribine on BVL over time [54]. However, in the CLARITY multicenter trial, there was a statistically significant annualized BVL reduction with cladribine administration in RRMS compared to placebo between months 6 and 24 [55]. Due to the long-lasting immunosuppressive effect of cladribine, lymphopenia is a known risk of its long-term use [56]. Other studies are still being conducted to better determine the effects of cladribine and the development of other types of malignancies.

## 7. Injectable Disease Modifying Therapies

### 7.1. Glatiramer Acetate (GA)

Glatiramer acetate, commercially known as Copaxone, is an FDA-approved injectable DMT for RRMS. Copaxone has demonstrated both tolerability and clinical maintenance of MS with long-term administration [57]. Its mechanism of action is complex. It is currently thought that GA acts by inhibiting myelin-specific T cells, most likely occurring at the periphery near injection sites or neighboring lymph node regions [58]. Ziemssen, et al. observed that administration of GA may be linked to increased brain-derived neurotrophic factor (BDNF) production, allowing GA-specific activated T cells to have a greater neuroprotective effect in the nervous system. In trials, patients who were administered glatiramer acetate had significantly reduced the cumulative number of new T2-weighted lesions [59].

The effect of glatiramer acetate on brain atrophy is controversial. Whilst data from the extended, open-label follow-up of the US trial [60] seem to indicate that long-term treatment with GA might prevent the loss of brain parenchyma in relapsing-remitting MS patients, longitudinal data from the European-Canadian MRI trial [61] suggest that, over a short-term period of treatment, GA does not have a clear-cut impact on the decrease of brain volume. However, a delayed effect is likely [61]. Crescenzo, et al., showed a possible favorable effect of GA on gray matter, either by reducing the accumulation of cortical lesions and/or slowing down the progression of gray matter atrophy [62].

### 7.2. Interferons

Two types of interferon medications are available: interferon-beta 1a and interferon-beta 1b. Interferon-beta 1a types include medications commercially known as Avonex, Rebif, and Plegridy. Betaseron, Betaferon, and Extavia are the most common types of interferon-beta 1b. Both types of interferons are thought to act on T cells, but the cytokines they target differ, allowing for different cell cascades to be altered [63]. One study which examined the effect of subcutaneous interferon beta-1b (Betaseron) on MRI outcomes did not demonstrate any significant changes in brain atrophy in the treated group compared to placebo. In subgroup analysis, it was found that patients with more gadolinium-enhancing lesions tended to also have higher cerebral volume loss than patients whose baseline MRI showed non-active lesions, suggesting that higher inflammation contributes to the loss of brain tissue [64]. Another study observed some slowing in brain volume loss in patients on intramuscular interferon beta-1a (Avonex), but only after the second year of interferon administration [65]. In clinical trials, administration of interferons was associated with increased risks of humoral-mediated autoimmune or allergy conditions were observed, requiring more stringent monitoring [63].

## 8. Infusions

### 8.1. Alemtuzumab

Alemtuzumab, available as Lemtrada, is FDA-approved for aggressive RRMS patients. It is a monoclonal anti-CD52 antibody which depletes both T- and B-lymphocyte counts in the body, followed by their reconstitution [66]. Clinical studies with alemtuzumab have shown favorable MRI outcomes, maintaining low levels of BVL throughout the 5-year study period, even though most patients only received medication within the 2-year period. In CARE-MS, it was found that alemtuzumab significantly decreased the rate of BVL in RRMS patients in both year 1, year 2, and throughout the whole study period compared to subcutaneous interferon beta-1a [67,68]. In CARE-MS II, for RRMS with first-line treatment-refractory disease, decreased BVL was observed in year 1, year 2, and throughout the entire study period; however, the results were statistically significant only over the first two years of the study period [69]. In the CARE-MS II 5-year follow-up study, annualized BVL rate continued to decrease in year 3 compared to the core study and remained low in years 4 and 5 [70]. Development of autoimmune diseases, such as Hashimoto thyroiditis and idiopathic thrombocytopenic purpura (ITP), were observed risks in patients taking alemtuzumab [71].

### 8.2. Mitoxantrone

Mitoxantrone, commercially known as Novantrone, is FDA-approved as an infusion treatment for aggressive RRMS, SPMS, and PRMS. It is administered every three months. It acts as a powerful immunosuppressive agent by targeting macrophages, B lymphocytes, and T lymphocytes. A precise mechanism is still to be determined, but it is thought that mitoxantrone induces apoptosis of B lymphocytes to then lower the secretion of pro-inflammatory cytokines [72]. Although brain atrophy data are not available, mitoxantrone has a favorable effect on the development of gadolinium-enhancing and T2 lesions. In one study, MRI measures for both RRMS and SPMS suggest that long-term use of mitoxantrone is effective in reducing inflammation and overall disease activity in the brain. The imaging outcomes appeared to coincide with clinical outcomes of reduced relapse rates, but EDSS score worsened in 1/5 patients at year 3 during follow up The investigators emphasize that clinical outcomes do not always mirror radiological outcomes in every case, though imaging measures can sometimes be used to predict disease progression [73]. Observed risks of mitoxantrone include systolic cardiac dysfunction and acute myeloid leukemia (AML) [74].

### 8.3. Natalizumab

Natalizumab, commercially available as Tysabri, is FDA-approved to treat RRMS patients as a monthly infusion. Natalizumab is a monoclonal antibody that targets specific subunits on lymphocyte surfaces. It does not, however, appear to alter regulatory T-cell functioning, which is often seen with many other MS treatment options. As a result, fewer new enhancing lesions were observed after one month of treatment, demonstrating its promise in clinical settings [75]. Several studies showed a favorable effect of natalizumab on brain atrophy. In two pivotal clinical trials, the rate of BVL increased in the first year of treatment and then significantly decreased [76,77]. Several post-marketing trials demonstrated a beneficial effect on brain atrophy and cognition and showed the effect on BVL that occurred in the first year of treatment represents the “pseudoatrophy phenomenon,” and is mainly due to white matter changes related to natalizumab’s anti-inflammatory activity [78,79]. In a 5-year prospective study, Zivadinov, et al., demonstrated a significant reduction of BVL, not associated with the number of natalizumab infusions. Patients who received continuous monthly natalizumab infusions showed less BVL than patients who discontinued or stopped and restarted treatments [80]. In another placebo-controlled study, it was observed that in patients on natalizumab, fewer Gd+ lesions were converting to T1 hypointense lesions than the placebo group, possibly as a result of reduced lymphocyte migration and lower measures of axonal damage [81]. Long-term use of natalizumab can lead to a higher risk of developing PML or other opportunistic infections, such as urinary tract infections. Upon the first administration of natalizumab, it is also possible that there is an allergic reaction or signs of rejection by the patient [82].

### 8.4. Ocrelizumab

Ocrelizumab, commercially known as Ocrevus, is one of the more recently approved infusion treatments available for both RRMS and PPMS patients. Ocrelizumab is a monoclonal anti-CD20 antibody that targets B cells, one of the first of its kind to alter B lymphocytes rather than T lymphocytes. In as early as phase 2 trials, it was observed that the administration of ocrelizumab appeared to reduce both MRI and clinical disease progression [83]. Another clinical study observed a reduction in the number of new T2 lesions as well as newly enlarging T2 lesions in subjects receiving ocrelizumab compared to placebo, indicating reduced levels of neural inflammation [84]. In the ORATORIO study, BVL was significantly lower in PPMS patients on ocrelizumab from week 24 to week 120 compared to patients on placebo [85]. Results from the OPERA I and OPERA II trials for RRMS patients showed a significant protective effect on BVL in the ocrelizumab group rather than the interferon beta-1a group in the OPERA I trial, but not in the OPERA II [86]. The most common side effects include infusion reactions and an increased risk for developing opportunistic infections such as UTIs or respiratory infections [83].

**Figure 1:**
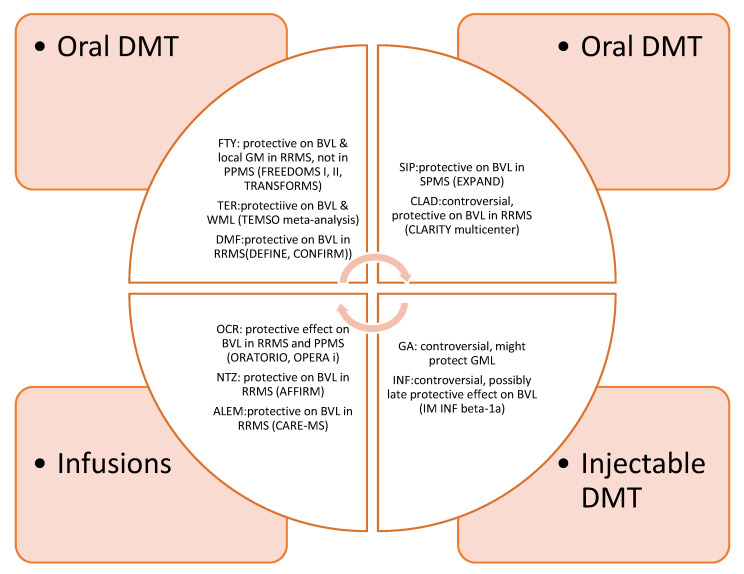
Effect of FDA-_approved DMT on brain atrophy. DMT: Disease modifying treatment. FTY: Fingolimod, TER: Teriflunomide, DMF: Dimethylfumarate, SIP: Siponimod, CLAD: Cladribine, GA: Glatiramer acetate, INF: Interferons, OCR: Ocrelizumab, NTZ: Natalizumab, ALEM: Alemtuzumab, WML: white matter loss, GML: gray matter loss, BVL: brain volume loss.

Overall, the effect of approved DMT is summarized in Figure 1.

## 9. Emerging Treatments

In addition to the available medication options for treating MS, new drugs that act on different biologic pathways are always being developed and improved upon. Below are medications that were recently approved or are under development.

1. Ozanimod: A recently FDA- approved selective S1P immunomodulator, not launched yet, showed very promising results in cognition and brain atrophy. In 2 phase III trials (SUNBEAM and RADIANCE) ozanimod slowed the rate of BLV, cortical grey matter, and thalamic volume compared to intramuscular interferon beta-1a [87].
2. Laquinimod: A novel oral immunomodulatory drug with a complex mechanism of action significantly decreased BVL versus placebo (ALLEGRO trial) and versus interferon beta-1a (BRAVO trial) in RRMS cases [88].
3. Bruton’s tyrosine kinase (BTK) inhibitors: A class of newer agents, the BTK inhibitors block the activation of B-lymphocytes both in vivo and in vitro [89]. This class includes fenebrutinib, ibrutinib, and evobrutinib; some of them are about to start entering multicenter trials and their effect on axonal integrity is currently unknown. A phase II trial of evobrutinib versus placebo showed a significant reduction in gadolinium-enhancing lesions with a non-significant effect on annualized relapse rate. Data on BVL were not reported [90].
4. Ibudilast: A phosphodiesterase inhibitor, originally used in asthma. In a phase II trial (SPRINT-MS) with progressive MS (both PPMS and SPMS) patients, it has shown a 48% difference in atrophy progression compared to placebo over 96 weeks [91].
5. Lipoic acid: A powerful anti-oxidant showed a very high impact on the annualized rate of whole-brain atrophy versus placebo in SPMS patients in a single-center study having BVL as the primary outcome [92].
6. High dose biotin: A member of the B-complex showed conflicting results in different clinical trials. An open-label pilot study showed reversal of disability at 9 months with a sustainable effect; however, the MRI data were disappointing and consistent with more new or enlarging lesions than the placebo group [93]. In a single-center study with SPMS, the biotin group showed worsening of disability versus placebo, despite stable MRI scans. More results are pending and atrophy data have not been reported as yet [94].
7. Cell-based therapies: Experimental therapies, with autologous hematopoietic stem cell transplantation (AHSCT) being the most known. AHSCT is in clinical trials and it might be a good option for patients with aggressive RRMS and pronounced inflammation [95]. Early administration and reduction of toxicity are important factors to be addressed before pursuing this therapeutic option [96]. It was observed that brain atrophy increased sharply in the first 2 years after treatment and decreased dramatically after the 2-year time point [97,98,99].
8. Aerobic exercise: Cardiorespiratory fitness was found to be associated with the preservation of gray matter volumes in the right post-central gyrus and midline cortical structures that are more commonly involved in MS, suggesting a protective role of high-intensity exercise on neurodegeneration [100].

## 10. Discussion

Inflammation and neurodegeneration are distinct entities, with overlapping features. Neurodegeneration is the major determinant of disability and its management is of utmost importance. It can be seen through all stages of MS, from disease onset to late stages of the disease. When neurodegeneration takes over as the main pathogenetic mechanism of MS, it is too late to reverse the damage. Therefore, early intervention to control inflammation and delay axonal injury and neuronal loss is needed.

FDA-approved disease-modifying agents have, at best, a modest effect on brain atrophy that is more prominent after the first year of treatment. The available DMTs mainly target the inflammatory components of the disease, but there is no DMT to completely halt or reverse axonal loss. The fact that DMTs with a similar mechanism (i.e., fingolimod and siponimod) have an opposite effect on progressive MS illustrates the modest benefit of anti-inflammatory agents on axonal injury, and the urgent need to develop molecules with a focus on neurodegeneration. Newer, emerging drugs such as the ibudilast may be very promising, and supplements such as lipoic acid are widely accessible, more affordable, and seem to have a higher impact than the traditional DMT; however, these findings need to be validated in multicenter phase III trials with large samples and long study duration. Exercise is less expensive and free of side effects with a potential beneficial effect on neurodegeneration; therefore, intense fitness training may boost the effect of DMT on BVL. 

There are still several developing ideas for how to produce an effective MS treatment, such as identifying new targets and further investigating the role of microglia, natural killer cells, and intrathecal IgG synthesis. It is well known that IgG synthesis occurs early in MS and once established, it persists and never disappears. Intravenous natalizumab and steroids cause only a small decrease the intrathecal IgG synthesis; however, it remains essentially unchanged [101].

Effective distribution of a novel MS treatment through the central nervous system is of paramount importance. Intrathecal administration of rituximab in SPMS patients was tried in the past with unsatisfactory results, demonstrating inadequate depletion of B cells in the CNS and persistence of the axonal damage marker, the neurofilament light chain [102]. Despite its significant drawbacks, intrathecal administration, either directly by pumps or indirectly through endogenous receptor-mediated transporters, is feasible and may play an important role in treating PPMS by more easily passing through the blood-brain barrier [103]. Small molecules, such as BTK inhibitors, have a higher blood-brain penetration and therefore easier administration. These inhibitors, especially those that target B-cells, may provide a more economical, convenient, and effective treatment option [89].

Within recent years, our knowledge about the basic mechanisms that may be responsible for axonal injury and loss is rapidly increasing. A better understanding of the mechanisms driving neurodegeneration will contribute to the development of novel therapies that may further delay, eliminate, or even reverse neurodegeneration. Novel and effective treatment strategies to prevent or even reverse brain atrophy is not only a priority, but also a necessity for the near future. At the same time, it represents one of the biggest challenges in the therapeutic management of MS patients. Further studies attempting new routes of administration and new drug formulas will lead to a greater understanding of neurodegeneration as it occurs in multiple sclerosis and how it can be mitigated.

## Figures and Tables

**Table 1:** Mechanism of action of FDA-approved DMT.

DMT	Commercial Name	Approved Indication	Proposed Mechanism (If Available)	Route of Administration
Dimethyl fumarate	Tecfidera	CIS, relapsing MS	Changes cytokine balance by inhibiting NrF2	Oral
Fingolimod	Gilenya	CIS, relapsing MS	Sphingosine-1-phosphate receptor modulator	Oral
Siponimod	Mayzent	CIS, relapsing MS (including active SPMS)	Sphingosine-1-phosphate receptor modulator (1 and 5)	Oral
Teriflunomide	Aubagio	CIS, RRMS	Mitochondrial dihydro-orotate dehydrogenase (DHODH) inhibitor	Oral
Cladribine	Mavenclad	Relapsing MS (including active SPMS)	Synthetic deoxyadenosine analogue, long lasting lymphocyte depletion	Oral
Glatiramer acetate (GA)	Copaxone	CIS, RRMS	Promotes anti-inflammatory response	Injectable
Interferons	Avonex, Rebif, Plegridy, Betaseron, Betaferon, Extavia	CIS, RRMS	Changes cytokine balance, favors anti-inflammatory cytokines	Injectable
Alemtuzumab	Lemtrada	Aggressive RRMS	Monoclonal antibody, anti-CD52, long lasting lymphocyte depletion	Infusion
Mitoxantrone	Novantrone	Aggressive RRMS, SPMS, and PRMS	Immunosuppressant (type II topoisomerase inhibitor)	Infusion (every 3 months)
Natalizumab	Tysabri	RRMS	Monoclonal antibody; limits T cell transmigration through the blood brain barrier	Infusion (every month)
Ocrelizumab	Ocrevus	RRMS and PPMS	Monoclonal antibody, anti-CD20, depletes B cells	Infusion

PPMS: primary progressive MS, SPMS: secondary progressive MS, PRMS: progressive relapsing MS, RRMS: relapsing remitting MS, CIS: clinically isolated syndrome, DMT: disease modifying treatment
